# The Effects of Slow-Release Dexamethasone in the Treatment of Diabetic Macular Edema

**DOI:** 10.3390/pharmaceutics17020174

**Published:** 2025-01-30

**Authors:** Enzo Maria Vingolo, Simona Mascolo, Lorenzo Casillo, Mattia Calabro

**Affiliations:** University La Sapienza of Rome, Sense Organs Department, Unit of Ophtalmology, Polo Pontino-Ospedale A. Fiorini, 04019 Terracina, Italy; enzomaria.vingolo@uniroma1.it (E.M.V.); lorenzo.casillo@uniroma1.it (L.C.); mattia.calabro@uniroma1.it (M.C.)

**Keywords:** diabetic macular edema, dexamethasone, microperimetry, mean retinal sensitivity

## Abstract

**Objectives:** to evaluate the efficacy of 0.7 mg dexamethasone intravitreal implant in the treatment of patients with diabetic macular edema through mean retinal sensitivity (MRS), best corrected visual acuity (BCVA), central retinal thickness (CRT) and fixation stability. **Methods:** patients (n = 50) with DME, best corrected visual acuity (BCVA) of 0.1 logMAR, and central retinal thickness (CRT) of ≥300 μm determined by optical coherence tomography were treated with 0.7 mg slow-release dexamethasone, and endpoints were evaluated one and three months after the injection. **Results:** The best corrected visual acuity, BCVA, whose mean values at baseline were 0.42 logMAR, improved significantly post dexamethasone injection, with mean values of 0.20 logMAR at one month and 0.24 logMAR at three months. The mean central retinal thickness, CRT, was 463 µm at baseline increasing to 297 µm at one month, and 315 µm at three months. Mean retinal sensitivity (MRS) was 12.31 dB at baseline. In line with other parameters, MRS also showed significant improvement at one month after slow-release dexamethasone treatment, with a mean value of 15.35 Db and the improvement was sustained at three months after injection, with a mean value of 14.71 dB. Fixation stability was assessed using the area of the third BCEA ellipse. At baseline, patients had an ellipse area of 53.68 degrees. At one month after injection, patients showed an improvement, with a mean ellipse area of 5.23 degrees, which was maintained at three months, with a mean ellipse area of 4.13 degrees. **Conclusions:** The dexamethasone implant of 0.7 mg met the efficacy endpoint for improvement in MRS, BCVA, CRT and fixation stability.

## 1. Introduction

Diabetic macular edema (DME) is a major complication of diabetic retinopathy and a leading cause of vision loss in diabetic patients worldwide. It is a retinal thickening that involves or is located near the center of the macula, and it is characterized by the accumulation of extracellular fluid in the macula, resulting from the breakdown of the blood–retinal barrier and increased vascular permeability. The global prevalence of diabetes is rising, and with it, the incidence of DME is also expected to increase significantly. According to Yau et al. (2012), the prevalence of DME among individuals with diabetes is estimated to be 6.81% globally [1]. Studies have shown that without timely treatment, DME can lead to progressive visual impairment and blindness [2]. Several risk factors contribute to the development of DME, including chronic hyperglycemia, hypertension, and the duration of diabetes [3]. Advances in diagnostic imaging, particularly optical coherence tomography (OCT), have revolutionized the detection and monitoring of DME by allowing detailed visualization of retinal structures [4]. The management of diabetic macular edema (DME) has evolved significantly over the past decade, with several effective therapeutic options now available. The primary goal of treatment is to reduce macular fluid accumulation and preserve or improve visual function.

The first-line treatment for DME currently involves intravitreal injections of anti-vascular endothelial growth factor (anti-VEGF) agents. These drugs, including ranibizumab, aflibercept, and bevacizumab, inhibit VEGF, a key factor that promotes increased vascular permeability and leakage in DME. A study by Wells et al. (2016) comparing the three anti-VEGF agents indicated that aflibercept may provide superior visual outcomes in patients with worse baseline visual acuity [5]. For patients who do not respond adequately to anti-VEGF therapy or who are unsuitable for such treatments, corticosteroids represent an alternative therapeutic option, since chronic inflammation is one of the major causes of edema. It develops due to prolonged hyperglycemia, which leads to oxidative stress, increased production of pro-inflammatory cytokines, such as IL-6 and TNF-α, and endothelial dysfunction. These processes result in heightened vascular permeability, fluid accumulation in the macula, and damage to retinal tissue. Intravitreal corticosteroid implants, such as dexamethasone and fluocinolone acetonide, are effective in reducing inflammation and vascular leakage. Studies have shown that corticosteroid implants can significantly reduce macular edema and improve vision, particularly in patients with chronic DME [6]. In some cases, focal or grid laser photocoagulation may still be employed, particularly in patients with focal DME. Although less commonly used as monotherapy since the advent of anti-VEGF agents, laser treatment can be combined with pharmacologic therapies to provide additional benefit in select cases. The Early Treatment Diabetic Retinopathy Study (ETDRS) demonstrated that laser photocoagulation reduces the risk of moderate visual loss in patients with DME [7]. Of all these therapeutic choices, slow-release dexamethasone, thanks to the presence of a biodegradable polymer matrix, allows it to degrade slowly in the eye without the need for surgical removal. Pharmacokinetic studies have shown that the concentration of dexamethasone in the retina and vitreous humor reaches a plateau within a few days of administration, maintaining a high concentration for two months, and then decreasing in the following months [8].

The most common adverse reactions to the use of slow-release dexamethasone in patients with diabetic macular edema include increased intraocular pressure and the development of cataracts [9]. In clinical practice, for the follow-up of diabetic macular edema following treatment with a slow-release intravitreal dexamethasone implant, both morphological and functional responses are taken into consideration. Regarding morphological response, it is now well-established that assessment through optical coherence tomography (OCT) can be used with great benefit, both in the diagnosis and management of diabetic macular edema [10]. Regarding functional response, it is generally evaluated in terms of best corrected visual acuity (BCVA). While necessary, BCVA evaluation as a functional parameter is not exhaustive, as it does not provide complete indications of various aspects of macular function. Also, it does not reveal visual system efficiency, particularly considering that visual acuity deterioration results from involvement of the macular center, a phenomenon not always occurring in the natural history of the disease. Thus, to more thoroughly investigate visual function, microperimetry has been adopted, providing detailed information on the degree and pattern of macular alteration compared to BCVA assessment alone. These considerations have led us to complement morphological evaluation with OCT of diabetic macular edema following slow-release dexamethasone treatment and BCVA assessment with functional evaluation using microperimetry. Microperimetry, or fundus-related perimetry, is a non-invasive diagnostic test that combines color fundus photography and computerized perimetry. Color fundus photography is the photographic representation of the ocular fundus, while computerized perimetry is a functional test that allows for mapping the visual field. In other words, microperimetry allows for the simultaneous evaluation of both morphological and functional data [11].

The primary objective of our study is to evaluate the functional response to dexamethasone treatment using microperimetry, focusing on pre-injection mean retinal sensitivity (MRS), one month, and three months post-injection. The secondary objectives of our study include assessing fixation stability, best corrected visual acuity (BCVA), and central retinal thickness (CRT) during the same period. The expected outcomes are improvements in these parameters following the injection, one month and three months after intravitreal dexamethasone implantation.

## 2. Materials and Methods

This study involved 50 eyes of as many patients (27 female and 23 male). The follow-up period for each patient lasted 3 months. Two patients were lost during the follow-up period. All participants had a diagnosis of type 2 diabetes mellitus with an average disease duration, defined as the time from initial diagnosis to the patient’s preliminary evaluation, of 5.53 ± 2.16 years. The average age of the patients was 64.32 ± 12.24 years. None of the treated patients had previously received intravitreal treatment. All patients were recruited from the Ophthalmology Unit at “Fiorini” Hospital in Terracina (LI) (Sapienza, University of Rome) between January 2019 and May 2020. All patients were informed of the study’s purposes and provided informed consent. The study adheres to the principles of the Helsinki Declaration.

Inclusion criteria for the study were as follows:

- Age over 30 years;
- Instrumentally diagnosed diabetic macular edema;
- BCVA of at least 0.1 logMAR at the time of the first visit;
- Retinal thickness > 300 µm at the first visit measured by spectral-domain OCT;
- Pseudophakic patients with uncomplicated surgeries more than one year prior to recruitment.

Exclusion criteria were:

- Structural damage within 500 µm from the fovea documented by spectral-domain OCT involving retinal layers that could preclude functional recovery and thus compromise study results. Specifically, the patients who showed the following structural lesions were excluded: absence of rarefaction of the junction between inner and outer segments of photoreceptors (IS/OS junction), retinal pigment epithelium (RPE) atrophy, subretinal fibrosis, alteration of the retinal nerve fiber layer from any previous or ongoing pathology, or the presence of large hard exudate plaques;
- Previous foveal laser treatments (grid or focal treatments);
- Any type of surgery or laser treatment performed in the six months prior to recruitment;
- History of chronic or acute inflammatory ocular diseases unresolved within six months prior to recruitment;
- Previous intravitreal therapy with steroids or anti-VEGF;
- Patients diagnosed with glaucoma or known to be steroid responders;
- Pseudophakic patients with a history of postoperative Irvine–Gass syndrome or posterior capsule rupture;
- Patients with central retinal vein occlusion or venous branch occlusion.

All study patients received treatment in the form of intravitreal injection of 0.7 mg dexamethasone using a preloaded disposable 22-gauge injector via pars plana. All implants were performed under sterile conditions in the operating room under optical microscope guidance. After the procedure, all patients were treated with topical antibiotics for 7 days following intravitreal implantation.

Evaluations performed for each patient included:

- Comprehensive ophthalmologic examination;
- Measurement of best corrected visual acuity using the ETRDRS chart;
- Applanation tonometry using a Goldmann tonometer slit lamp biomicroscopy;
- Complete ophthalmoscopic examination including examination of the posterior pole using a slit lamp and examination of the retinal periphery using a binocular indirect ophthalmoscope;
- OCT examination using SD-OCT (Spectralis Heidelberg, Germany) with FAST grid for studying the entire posterior pole and central retinal thickness (CRT) reconstruction and study program of the nerve fiber layer;
- Microperimetry to assess retinal sensitivity and fixation stability through a 30 s examination.

A microperimeter MP-1 (Nidek, Padua, Italy) was used with a 12° macula program, 10 dB, Goldmann III stimuli for 200 msec presented randomly on a white monochromatic background covering the central 10° of the retina.

Statistical analysis was performed using Prism version 9.5.0 (GraphPad Software Inc., San Diego, CA, USA). Data were expressed as mean standard deviation (SD). The Anderson–Darling and Kolmogorov–Smirnov tests were applied to assess whether data were normally distributed. Data analysis used the Wilcoxon signed-rank test and Student’s *t*-test on an Excel spreadsheet. A *p* value of less than 0.05 was considered statistically significant. Each variable was independently evaluated without being correlated with others. However, for the interpretation of this study, it should be taken into account that the results demonstrated considerable variability, particularly for BCEA and MRS, while CRT demonstrated more moderate variability.

## 3. Results

For this study, 50 eyes of as many patients (27 women and 23 men) with type 2 diabetes mellitus were recruited. Two patients were lost to follow-up. The mean age of the patients was 64.32 ± 12.24 years. The mean duration of diabetes, defined as the time from initial diagnosis to the preliminary evaluation, was 5.53 ± 2.16 years.

The mean values at baseline, before dexamethasone injection, were as follows: BCVA 0.42 ± 0.18 logMAR; CRT of 463 ± 180 μm; MRS of 12.31 ± 5.2 dB; ellipse area encompassing 99.4% of fixation points (III BCEA ellipse) of 53.68° ± 82.57°.

### 3.1. Best Corrected Visual Acuity

The best corrected visual acuity, BCVA, whose mean values at baseline were 0.42 ± 0.18 logMAR, showed a statistically significant improvement (*p* < 0.05) at one month post dexamethasone injection, with mean values of 0.20 ± 0.16 logMAR. This improvement partially persisted at three months post-injection, with stable mean values of 0.24 ± 0.14 logMAR.

### 3.2. Central Retinal Thickness

**Figure 1:**
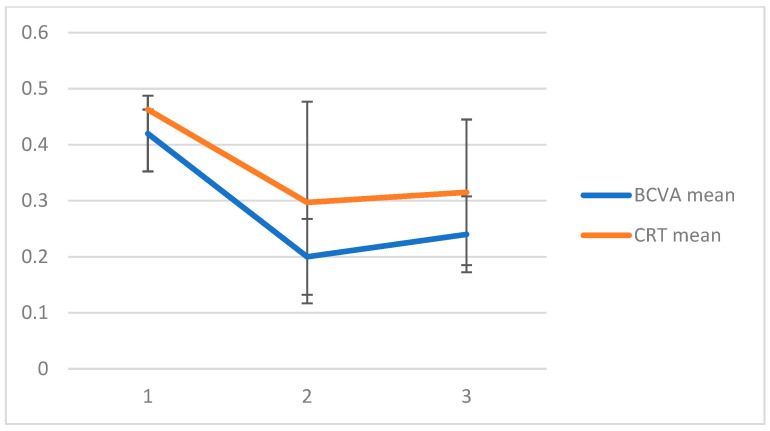
BCVA mean ± SD and CRT mean ± SD at baseline, at one month and at three months after the injection.

The mean central retinal thickness, CRT, was 463 ± 180 µm at baseline. CRT showed significant improvement (*p* < 0.05) at one month after slow-release dexamethasone treatment, with a mean value of 297 ± 130 µm. Similarly to BCVA, at three months after treatment, the improvement was maintained, with a mean value of 315 ± 120 µm. The data for BCVA and CRT are summarized in the Figure 1.

### 3.3. Mean Retinal Sensitivity

Mean retinal sensitivity (MRS) was 12.31 ± 5.2 dB at baseline. In line with other parameters, MRS also showed significant improvement (*p* < 0.05) at one month after slow-release dexamethasone treatment, with a mean value of 15.35 ± 5.5 dB. The improvement was sustained at three months after injection, with a mean value of 14.71 ± 5.4 dB.

### 3.4. Fixation Stability

**Figure 2:**
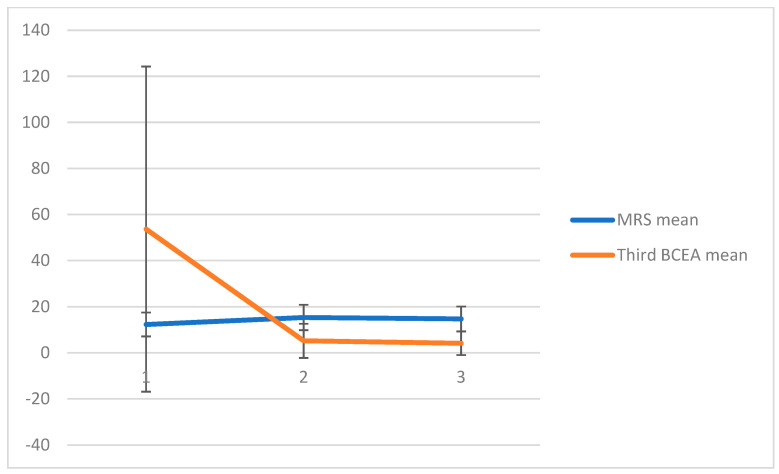
MRSA mean ± SD and Third BCEA mean ± SD at baseline, at one month and at three months after the injection.

Fixation stability was assessed using the area of the third BCEA ellipse, which includes 99.4% of fixation points. At baseline, patients had an ellipse area of 53.68 ± 82.57 degrees. At one month after injection, patients showed a statistically significant improvement (*p* < 0.05), with a mean ellipse area of 5.23 ±7.41 degrees. At three months after slow-release treatment, the mean ellipse area was 4.13 ± 5.1 degrees. The data for MRS and Third BCEA ellipse are summarized in the Figure 2.

The mean values of the data obtained from the sample in terms of improved best corrected visual acuity (BCVA), reduced central retinal thickness (CRT), improved mean retinal sensitivity (MRS), and improved fixation stability, evaluated before injection and at one month and at three months after injection, are summarized in Table 1.

## 4. Discussion

In recent decades, multiple studies have evaluated the efficacy and safety of the intravitreal slow-release dexamethasone implant, often through comparisons with other intravitreal drugs used in the treatment of diabetic macular edema, such as anti-VEGF medications [12,13]. Before slow-release dexamethasone was approved for diabetic macular edema, studies had demonstrated its effectiveness in treating macular edema secondary to branch retinal vein occlusion or central retinal vein occlusion, as well as in therapy for non-infectious posterior segment inflammation of the eye. Indeed, it was only more recently, in 2014, that dexamethasone injection was approved by the FDA for the treatment of diabetic macular edema in adult patients.

In our prospective study, we investigated the impact of intravitreal slow-release dexamethasone implantation on macular morphology and function in 50 eyes of as many patients with diabetic macular edema over a follow-up period of three months. The decision to evaluate the effects of intravitreal dexamethasone implantation during this observation period was driven by the desire to observe outcomes following a single injection, within a timeframe where additional intravitreal injections are not typically administered. Moreover, in the majority of studies in the literature, improvements were observed within the six months following dexamethasone implantation. Understanding the pharmacokinetics involved is crucial in interpreting the outcomes observed in our sample. In a study by Chang-Lin et al., it was observed that dexamethasone reaches peak concentration in the retina and vitreous at day 60 post-injection, coinciding with the fragmentation of most of the dexamethasone implant. Beyond day 60, the remaining dexamethasone fragments continue to slowly degrade, releasing dexamethasone from the remaining material without a final burst of drug release [8]. This observation correlates well not only with our study findings but also with those reported in other studies in the literature. Indeed, all studies known to us evaluating the response to slow-release dexamethasone treatment show significant and sustained improvement in mean visual acuity observed within the first three months and partially maintained over the subsequent six months following intravitreal implantation. Several studies have demonstrated the efficacy of dexamethasone implants in managing DME. The MEAD study (Macular Edema: Assessment of Implantable Dexamethasone in Diabetes) was a pivotal phase III clinical trial that evaluated the safety and efficacy of the dexamethasone implant in patients with DME. The study found that repeated dexamethasone implant injections led to significant improvements in visual acuity and reductions in central retinal thickness compared to placebo over a three-year period [9]. Specifically, 22.2% of patients treated with the dexamethasone implant (0.7 mg) gained 15 or more letters of vision, compared to 12% in the placebo group. In the BEVORDEX study, which compared intravitreal bevacizumab (an anti-VEGF agent) with dexamethasone, it was shown that both therapies were effective in reducing DME, but patients in the dexamethasone group experienced more rapid improvements in retinal thickness [14]. However, bevacizumab showed better visual outcomes over a longer period.

Morphological analysis of the macula was performed using OCT to measure central retinal thickness. Visual function was investigated through BCVA and, innovatively, compared to much of the literature, through microperimetry, providing information on retinal sensitivity and fixation. The results from our study sample showed significant improvement in the parameters considered, both morphologically, in terms of central retinal thickness, and functionally, in terms of BCVA and retinal sensitivity and fixation.

In the overall evaluation of patients with diabetic macular edema, especially following intravitreal steroid drug administration, evaluating treatment effects cannot disregard assessment of both the morphological and functional status of the retina.

In clinical practice, as well as in the literature, functional assessment primarily considers best corrected visual acuity (BCVA), which indicates the best possible vision a patient can achieve with corrective lenses, if needed. Morphological assessment, on the other hand, is generally obtained through OCT evaluation, which objectively measures retinal thickness in micrometers and evaluates changes in intra-retinal fluid following treatment. Regardless of specific pharmacological treatment for diabetic macular edema, patients with this condition have been the subject of numerous studies that highlight the need not to rely solely on BCVA to fully describe diabetic patients’ functionally. For this reason, microperimetry has been proposed as a valuable tool for comprehensively assessing the functional pattern of patients with diabetic macular edema.

As previously mentioned, BCVA remains the standard evaluation for diabetic patients in clinical practice, but it primarily explains foveal macular function. In contrast, functional evaluation through microperimetry, as conducted in our study, allows for a more accurate assessment of topographic sensitivity across the entire central retinal field, providing valuable additional insights. Moreover, microperimetry allows for an analysis of the association between retinal sensitivity and the retinal state of the corresponding area, by simultaneously obtaining functional and morphological data through fundus photography. Another significant advantage of microperimetry is the ability to conduct follow-ups based on objective and reproducible data.

Several studies on diabetic macular edema have emphasized that there is often a mismatch between a patient’s visual acuity and the severity of diabetic retinopathy, and how patients often experience visual disturbances such as blurring and relative scotomas, and especially decreased contrast sensitivity, which are not always correctly evaluated and quantified in routine exams [15]. Furthermore, it has been shown in various studies that even diabetic patients with relatively good visual acuity may have reduced reading speed. Indeed, it has been established through several studies that there is an actual discrepancy between the microanatomy of diabetic patients with retinal macular edema and their visual function. These evaluations effectively underscore the inadequacy of BCVA as a parameter to comprehensively describe the functional deterioration caused by diabetic macular edema [16], and consequently the response to treatment. Moreover, this parameter alone is inadequate to fully describe the impact that diabetic macular edema may have on the patient’s quality of life. Nonetheless, BCVA remains an essential and irreplaceable parameter in the evaluation of diabetic patients, especially in clinical practice, and also for logistical reasons.

Some studies, using microperimetry in diabetic patients, have shown that in cases of diffuse diabetic macular edema, there is poor correspondence between visual acuity and retinal light sensitivity, whereas in the context of focal alterations, such as hemorrhages, there is a reduction in both visual acuity and light sensitivity. This could be explained by the topography of the two different types of lesions, as diffuse macular edema displaces cells and scatters light, while dense focal alterations block light arrival to photoreceptors before structural damage occurs [15].

The introduction of OCT into clinical practice for evaluating patients with diabetic macular edema has revolutionized its treatment. Indeed, OCT is now the most widely used tool, especially since it allows for quantitative measurement of macular thickness and evaluation of macular morphology [17]. With only modest correlation between visual acuity and central retinal thickness [18], the evaluation of both parameters is essential, and some have proposed using biomarkers other than central retinal thickness for morphological evaluation of patients with diabetic macular edema.

Based on these considerations, we decided to comprehensively evaluate patients with diabetic macular edema in our prospective study. The primary endpoint of this study was to evaluate mean retinal sensitivity using microperimetry before dexamethasone intravitreal injection, at one month and at three months post-intravitreal injection. Concurrently, over the same observation period and timings, we evaluated fixation stability, again using microperimetry, BCVA, and central retinal thickness using OCT.

Regarding best corrected visual acuity (BCVA), the data obtained from our sample demonstrated a considerable improvement compared to the values patients presented at baseline. Indeed, study participants, who had an average baseline BCVA of 0.42 ± 0.18 logMAR, achieved statistically significant improvement, averaging 0.24 ± 0.14 logMAR at three months post-intravitreal injection. These findings are entirely consistent with those found in the literature thus far. BCVA is indeed a standard parameter used in the evaluation of diabetic patients, and in clinical practice, as well as in the literature, it is used to assess the functional effects of dexamethasone treatment. A similar consideration can be made regarding central retinal thickness, assessed using OCT. The data obtained from the analysis of our sample show statistically significant improvements in all patients treated with slow-release dexamethasone. The average central retinal thickness of patients was 463 ± 180 μm at the baseline preliminary visit before injection. This is practically reduced by just under one-third at three months post-intravitreal injection, with an average value of 315 ± 120 μm.

The BCVA and central retinal thickness assessed through OCT in the context of our study are consistent with the results presented in the literature. Indeed, these two parameters are among the most commonly used to assess the functional and morphological status of the response to slow-release dexamethasone treatment. The three-year randomized clinical trial conducted by the MEAD study group compared the efficacy and safety of dexamethasone 0.17 mg and 0.35 mg versus placebo treatment in a large population of patients with diabetic macular edema. This study revealed that up to one-third of patients treated showed significant improvement in BCVA, with an increase of at least 15 letters. Moreover, this study also considered central retinal thickness, which was reduced by an average of 110 μm compared to baseline. Both outcomes were considerably better following treatment with slow-release dexamethasone 0.7 mg rather than 0.35 mg [9]. The PLACID study, a randomized study in patients with diffuse macular edema, compared treatment with dexamethasone 0.7 mg and laser photocoagulation to treatment with laser photocoagulation alone. This study showed statistically greater improvements in baseline BCVA in the group receiving dexamethasone and photocoagulation up to 5 months, with improvement also observed in retinal thickness and vascular leakage area [19].

The most innovative aspect of our study compared to most studies aiming to comprehensively evaluate patients with diabetic macular edema following intravitreal treatment with slow-release dexamethasone is the study of visual function using microperimetry. The use of this tool allows for the investigation of retinal sensitivity and fixation, two aspects sometimes overlooked in the outpatient evaluation of diabetic patients, but which can be extremely useful, especially in terms of their utility in potential visual rehabilitation of visually impaired patients.

The results obtained from our study sample showed significant improvements in both parameters: fixation stability and mean retinal sensitivity. Regarding mean retinal sensitivity, the results confirmed the expected outcome, showing an improvement from baseline values of 12.31 ± 5.2 dB to 14.71 ± 5.4 dB three months post-injection treatment.

Most studies in the literature have assessed the functional aspect of patients with diabetic macular edema following treatment with slow-release dexamethasone by relying solely on the BCVA parameter. However, the few studies that have also utilized microperimetry in functional evaluation have shown results fully consistent with those obtained in our study. Mastropasqua et al. evaluated the morphology and function of patients with diabetic macular edema treated with dexamethasone over a one-year follow-up period, using microperimetry and OCT. Their results also showed significant improvements in BCVA and macular sensitivity within just one month of treatment, and this effect persisted for the following five months. The average retinal thickness in their study similarly showed a significant reduction in the first month and remained stable at lower values during the next four months, with no significant adverse effects. Additionally, they observed an inverse correlation between central macular thickness and retinal sensitivity in most patients six months post-treatment, confirming the importance of edema reduction for improving retinal visual function [20]. Although our study did not explicitly relate the observed variables, it is clear from our data that there is also an inverse correlation between central retinal thickness and functional data. Studies in the literature investigating retinal sensitivity in response to slow-release dexamethasone treatment in patients with diabetic macular edema are quite scarce. However, Querques et al. evaluated the impact of dexamethasone injection on the morphology and macular function of patients with diabetic macular edema secondary to central retinal vein occlusion using microperimetry. Their study demonstrated significant improvements in BCVA, central macular thickness assessed by OCT, and retinal sensitivity assessed by microperimetry, one-month post-treatment. Furthermore, this study correlated retinal sensitivity with central macular thickness, showing a negative correlation between the two. In other words, even in macular edema following central retinal vein occlusion, lower retinal thickness is associated with better retinal sensitivity and, consequently, better visual performance [21].

An interesting study on retinal sensitivity was conducted by Vujosevic et al., who investigated changes in retinal sensitivity, assessed using microperimetry, and macular thickness in patients with clinically significant and non-significant diabetic macular edema. Based on their data, they hypothesized that macular sensitivity is one of the most prognostically important parameters in the outcome of treatment for diabetic macular edema. Indeed, the retinal sensitivity map provides a wealth of information on central macular function as it can document each specific area where macular function is altered. Moreover, the importance of being able to automatically follow the same retinal area during patient follow-up should not be underestimated, as it allows for detailed monitoring of retinal function and documentation of scotomatous areas, which may not alter the patient’s visual acuity but undoubtedly causes visual disturbances [22].

In summary, based on the analysis of data obtained from our study, in which patients with diabetic macular edema who had not previously received any intravitreal treatment were treated, the final result is a statistically significant improvement in all examined variables: BCVA, central retinal thickness, fixation stability, and retinal sensitivity three months after intravitreal slow-release dexamethasone implantation, confirming the expected outcomes. During the observation period, none of the study participants reported adverse effects potentially caused by the implant, such as increased intraocular pressure or glaucoma. Unfortunately, the data collected in this study exhibited considerable variability, particularly concerning the MRS and BCEA ellipse values. This variability may represent an additional limitation of the study, as it could introduce uncertainty in the interpretation of the results. Such variability may be attributed to factors such as inherent biological differences among participants or the complexity of the data itself. A more detailed exploration of these variations could help refine the understanding of the underlying patterns and improve the robustness of future analyses.

Other limitations of our study include the number of patients enrolled and the observation period, which, if extended, could have provided more information on the potential stability or improvement of the obtained results, potentially also evaluating the effect of further slow-release dexamethasone injections.

## 5. Conclusions

Diabetic retinopathy remains one of the leading causes of blindness in the Western world. Our study, which evaluates the effects one month and three months after Ozurdex injection in naive patients with diabetic macular edema, is one of the few studies known to us that investigates patients with diabetic macular edema treated with dexamethasone using microperimetry, specifically to assess retinal sensitivity. The primary goal of our study was to investigate whether, following treatment with Ozurdex, patients would show an improvement in mean retinal sensitivity, and the results obtained from our study sample confirmed the expected outcome. Indeed, in our study, we observed a significant improvement in all parameters examined: retinal sensitivity, fixation stability, BCVA, and central retinal thickness.

Beyond the research objective and the intention to investigate the effect of intravitreal dexamethasone implantation, this study can be useful in highlighting the role that microperimetry plays in evaluating diabetic patients. Very often, in fact, during outpatient visits, the aspect of retinal sensitivity in patients with diabetic macular edema is overlooked. However, recent studies that have investigated this parameter, even for evaluating macular edema associated with other retinal diseases, increasingly emphasize its importance.

## Figures and Tables

**Table 1:** Synthesis of the obtained data.

Parameter	Baseline	at One Month from Injection	at Three Months from Injection
BCVA mean (SD)	0.42 (±0.18) logMAR	0.20 (±0.16) logMAR	0.24 (±0.14) logMAR
CRT mean (SD)	463 (±180) mcm	297 (±130) mcm	315 (±120) mcm
MRS mean (SD)	12.31 (±5.2) dB	15.35 (±5.5) dB	14.71 (±5.4) dB
Third BCEA mean (SD)	53.68° (±82.57°)	5.23° (±7.41°)	4.13° (±5.1°)
